# Primary care use of antipsychotic drugs: an audit and intervention study

**DOI:** 10.1186/1744-859X-4-18

**Published:** 2005-11-29

**Authors:** Ann M Mortimer, Charles J Shepherd, Michael Rymer, Alison Burrows

**Affiliations:** 1 Foundation Chair in Psychiatry/Head of Department, The Department of Psychiatry, The University of Hull, Cottingham Road, Hull, HU6 7RX, UK; 2 Research Nurse, The Department of Psychiatry, The University of Hull, Cottingham Road, Hull, HU6 7RX, UK; 3 Pharmaceutical Advisor, Eastern Hull Primary Care Team, Central Office, Netherhall, Wawne Road, Sutton, UK, Hull, HU7 4YG, UK; 4 Consultant Psychiatrist, Harrogate District Hospital, Lancaster Park Road, Harrogate, North Yorkshire, HG2 7SX, UK

## Abstract

**Background:**

Concerns regarding the use of antipsychotic medication in secondary care suggested an examination of primary care prescribing.

**Aim:**

To audit and intervene in the suboptimal prescribing of antipsychotic drugs to primary care patients.

**Design of study:**

Cross-sectional prevalence: subsequent open treatment intervention.

**Setting:**

Seven of the 29 practices in the Eastern Hull Primary Care Trust.

**Methods:**

Criteria for best practice were developed, against which prescribing standards were tested via audit. Patients identified as suboptimally prescribed for were invited to attend an expert review for intervention.

**Results:**

1 in 100 of 53,000 patients was prescribed antipsychotic treatment. Diagnoses indicating this were impossible to ascertain reliably. Half the regimes failed one or more audit criteria, leaving diagnosis aside. Few practices agreed to patients being approached: of 179 invitations sent, only 40 patients attended. Of 32 still taking an antipsychotic drug, 26 required changes. Mean audit criteria failed were 3.4, lack of psychotic disorder diagnosis and problematic side effects being most frequent. Changes were fully implemented in only 16 patients: reasons for complete or partial failure to implement recommendations included the wishes or inaction of patients and professionals, and worsening of symptoms including two cases of antipsychotic withdrawal syndrome.

**Conclusion:**

Primary care prescribing of antipsychotic drugs is infrequent, but most is unsatisfactory. Intervention is hampered by pluralistic reluctance: even with expert guidance, rationalisation is not without risk. Use of antipsychotic drugs in primary care patients whose diagnosis does not warrant this should be avoided.

**How this fits in:**

This study adds to concerns regarding high levels of off-licence use of potentially harmful medication. It adds evidence of major difficulties in rationalizing suboptimal regimes despite expert input. Relevance to the clinician is that it is better to avoid such regimes in the first place especially if there is no clear 'exit strategy': if in doubt, seek a specialist opinion.

## Introduction

We have previously published on the utilization of high dose antipsychotic treatment and polypharmacy in secondary care, and lack of adherence to appropriate guidelines [25]. The publication of NICE guidance on antipsychotic treatment in schizophrenia [4] would, we assumed, result in positive changes in secondary care prescribing. This guidance recommended atypical antipsychotic drugs in many common clinical situations, including for new patients, relapsing patients and symptomatically well controlled patients if side effects were unacceptable: polypharmacy and high doses were advised against.

Given the unsatisfactory state of secondary care prescribing demonstrated by our first study, we considered that the situation in primary care may benefit from examination particularly in the context of NICE. We therefore set up a further audit to identify patients of general practitioners receiving potentially problematic antipsychotic regimes, with a subsequent optional intervention to be offered to these GPs and their patients to rationalize their medication. The overall aim was to improve the wellbeing of a large number of patients currently receiving antipsychotic treatment sub-optimally. Optimizing such medication regimes should, we anticipated, have the effect of minimizing symptoms and side effects while maximizing quality of life.

The Eastern Hull Primary Care Trust (PCT) agreed to support the audit. This PCT has a catchment population of 125,000, with a typical range of urban inner-city health & social problems. It comprises 29 practices including 57 GPs, 17 of them single handed. There were at the time of the audit 12 community pharmacists working with 23 of the practices, offering hands-on prescribing support. From 2000 to 2003, the total number of prescriptions for antipsychotic drugs in Eastern Hull PCT rose moderately from 12117 to 12703 per year: however their cost rose markedly, from £215,752 to £324,511. While the usage and cost of conventional and depot medications remained constant, the usage and cost of atypical antipsychotic drugs, particularly olanzapine and risperidone, increased substantially.

## Method

The following audit criteria were adopted to define possible suboptimal prescribing in the patient group. They were derived from the literature, and a process of discussion and consensus finding between the four authors.

1. On thioridazine [22]

2. On more than one antipsychotic drug [4]

3. Psychotropic polypharmacy (increased risks of side effects and interactions: evidence in support of efficacy unclear in many diagnostic categories)

4. Greater than recommended maintenance dose [4]

5. Dose less than a quarter of recommended maintenance dose (therefore dubious efficacy)

6. No current diagnosis indicating an antipsychotic i.e. psychosis or short-term behavioural disturbance [3]

7. Long term anticholinergic treatment [27]

8. Not reviewed by GP or psychiatrist for 1 year

9. Unresolved problematic symptoms

10. Unresolved problematic side effects

Community pharmacists working in GP practices attended a training session about the project and the audit criteria, run by AM and a research nurse. They then audited all patients prescribed any antipsychotic medication in 7 of the 29 practices in Eastern Hull PCT. Patients were identified through electronic patient records systems at the surgeries. Audit criteria for identified patients were checked using electronic records, longhand records and personal enquiry of the GP if necessary.

For the subsequent intervention study, GPs were asked for permission to invite patients identified as failing any audit criteria for an appointment with AM and CS. Participating surgeries were provided with the text of an invitation letter, to be printed out on surgery notepaper and sent to eligible patients by practice staff: this preserved patient anonymity. GPs were offered advice regarding their patients who failed to respond or refused to be seen. Patients agreeing to a review were notified to CS, who subsequently attended the surgery to examine their notes and summarized their history prior to an appointment with AM and CS. Patients were seen at the surgery or, if they preferred, at their home. When seen, patients were asked to provide written consent for AM and CS to administer ratings of symptoms, side effects, general function and quality of life. The current medication regime and the patients' general mental health and well-being were then discussed. Proposed changes in medication, if any, were shared with the patient, and written advice on those agreed was given. Patients were informed that a follow-up appointment would be sent to assess progress once the changes had been implemented. The GP was informed in writing of the evaluation, and asked to implement the recommendations regarding medication changes.

Symptoms were rated with the Brief Psychiatric Rating Scale (BPRS), which identifies a broad spectrum of psychopathology across diagnostic groupings [20]. Antipsychotic side effects were measured with the Abnormal Involuntary Movements Scale (AIMS) [2] which assesses Parkinsonism, dyskinesia and akathisia. Side effects were also enquired about in general terms with each patient. General function was assessed using the Global Assessment of Function (GAF) [9] and the Clinical Global Impression (CGI) [1]. Quality of life was measured with the Quality of Life Self-Assessment Scale (QLSAS) [24]. Basic demographic and clinical data were collected: age, sex and clinical diagnosis from GP notes and the interview. At follow-up after a clinically appropriate period, patients' general mental health was reviewed and their medication noted: the rating scales were repeated. Non-parametric Wilcoxon signed ranks tests were carried out in order to ascertain whether changes in medication were associated with any significant changes in rating scale scores.

## Results

Almost 53,000 general practice patients were screened by the community pharmacists: 1% were prescribed antipsychotic drugs. The most frequent reasons for audit criterion failure were psychotropic polypharmacy and chronic anticholinergic treatment. However, community pharmacists reported insurmountable difficulty in establishing the diagnosis of patients prescribed antipsychotic drugs by their GPs even when case notes were scrutinized and personal enquiries made of the GPs. This criterion therefore had to be abandoned as the majority of those prescribed antipsychotic treatment would have failed it. Similar caveats applied to the criteria regarding unresolved symptoms and side effects: no figures were returned, although all these criteria were examined in patients presenting for the subsequent intervention. Excluding unfeasible criteria, overall 280 i.e. just over half the patients were being prescribed regimes of medications which failed one or more audit criteria.

A minority of practices accepted the opportunity for review of their patients. 179 invitations to patients were sent: only 74 replies were received. We were informed later that 13 of the patients resided in a single nursing home: none replied. 54 patients accepted an appointment to be seen: 14 failed to attend, leaving 40 patients who underwent at least an initial evaluation. This represented only 23% of the number eligible for a review, whose GPs had agreed to their being approached.

The mean age of the patients was 59 years, with a range of 62 years: the oldest patient was 95 and the youngest patient was 33. 15 patients were men and 25 were women: there were no significant sex differences in age or any rating scale scores either initially or at follow-up. The diagnoses of these patients indicated that most were being prescribed antipsychotic medication off license. Clinically the diagnoses included 12 patients with uni-polar depression, 8 with learning disability, 6 with schizophrenia, 4 with anxiety or panic disorder and 3 with vertigo: 1 each dementia, personality disorder, bipolar disorder, alcohol dependence, obsessive-compulsive disorder and restless legs. In one patient no formal diagnosis could be arrived at even after careful scrutiny of her history and two personal interviews with both the patient and her mother.

**Figure 1:**
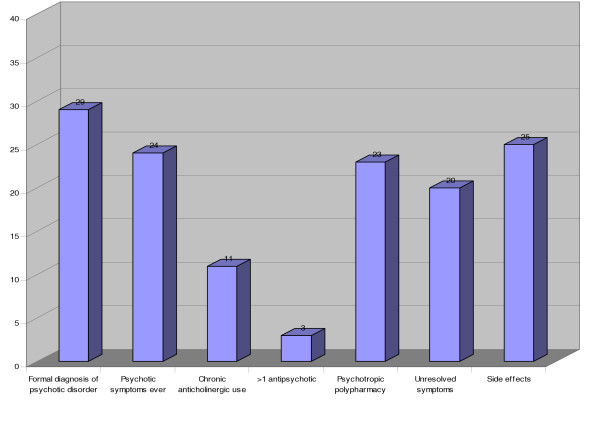
Failure of Audit Criteria.

32 patients were still taking antipsychotic treatment when seen. Figure 1 demonstrates the pattern of failure of audit criteria of these patients: our investigations revealed that 5 of the 29 with no formal psychotic disorder diagnosis did in fact have convincing evidence of psychotic symptoms either previously or currently. All the patients on more than one antipsychotic drug were diagnosed with schizophrenia. The mean number of criteria failed per patient was 3.4, with a range of 1–6: the standard deviation was 1.2.

Only 8 (25%) of the patients were prescribed atypical drugs, the rest were prescribed conventional treatment. Clinical actions were recommended for 26 out of the 32 patients remaining on antipsychotic treatment at the time of interview. In half of the patients [15], stopping antipsychotic treatment altogether was advised. All 11 patients taking anticholinergic drugs on a chronic basis were advised to cease them. Other psychotropic drugs were suggested to be discontinued in 8 patients, some of whom had already stopped antipsychotic treatment before the first interview. In only 5 of the 32 patients was an atypical antipsychotic treatment recommended instead of existing conventional treatment.

Rating scale scores demonstrated that the 26 patients whose prescribing required amendment were minimally or mildly symptomatic for the most part. However they had a significant burden of motor side effects, and their function was far from optimal (see Table 3). Patients experienced great difficulty in filling in the QLSAS: this scale comprises a comprehensive list pertaining to life in general e.g. utilities, housing, access to leisure etc. Patients were asked to mark items with which they were not satisfied. Patients did not appear to relate well to the items as stated, and frequently tried to mark all which were satisfactory, becoming confused when directed not to. This difficulty was not compensated for by the QLSAS's freedom from mood and side effect items, and its use had to be dispensed with.

**Table 3:** Rating scale scores at each interview, changes and significance over time

	Initial interview: n = 26			Follow-up interview: n = 24			p
	
	Mean score	range	Sd	Mean score	range	sd	
BPRS	7	1–17	4	5	0–12	3	0.006
CGI	2.8	0–6	1.4	2.6	1–6	1.3	ns
AIMS	10	0–39	10	7	0–27	7	0.001
GAS	61	10–93	22	66	15–95	20	ns

BPRS – Brief Psychiatric Rating Scale

CGI – Clinical Global Impression

AIMS – Abnormal Involuntary Movements Scale

GAS – Global Assessment Schedule

BPRS symptoms scores at second interview had improved to a statistically but not clinically significant degree. AIMS side effects scores had reduced significantly: CGI and GAS scores were improved, but this was not statistically significant. Although all 24 patients who attended follow-up were included in the analysis, a third, i.e. 8 patients had not altered their medication as advised, either partially or at all. 4 patients unfortunately felt worse on their new regimes than previously, and had reverted to their former prescriptions. These included 2 patients with definite and unpleasant conventional antipsychotic withdrawal syndromes. One patient decided herself not to make the changes after considering what had been advised: the CPN of another patient appeared to be the deciding factor in the continuation of the patient's suboptimal treatment, citing the consequences of relapse. In two patients the GP and consultant failed to alter the prescription for reasons of oversight.

## Illustrative cases

1 A 63 year old man with a 20 year history of chronic depression subsequent to a road accident (which caused several hours' loss of consciousness) and a one year history of epilepsy. He had mild depressive symptoms but no psychotic symptoms at any point. He was taking 75 mg chlorpromazine, 10 mg amitriptyline (originally for headache) and 5 mg nitrazepam daily. He had a marked tremor and complained of restlessness. He was advised to reduce and stop the chlorpromazine over six weeks on the grounds of tremor, probable akathisia, depressogenic and theoretical epileptogenic effects. When reviewed three months later, the patient described a severe exacerbation of restlessness, feeling hot, cold and sweating during his dosage reduction, to the point where his GP was obliged to reinstate the original dose. The patient's GP had substituted citalopram 20 mg for the 10 mg amitriptyline at our suggestion. The patient reported feeling more relaxed on this regime and furthermore had been able to stop using codeine for his headaches and laxatives for his previous constipation subsequent to codeine.

2 A 73 year old lady with diagnoses of mild learning difficulties and bipolar affective disorder, stable for the last three years and living in a nursing home. She was taking carbamazepine 300 mg bd, risperidone 2 mg bd, paroxetine 20 mg bd, and thyroxine. She was grossly obese with a BMI of 40, suffered from osteoarthritis and walked with a Zimmer frame. She also suffered from Parkinsonism and osteoporosis. We advised gradual alterations culminating in valproate semi-sodium as mood stabilising mono-therapy only. The grounds for this were the lack of tolerability and poor efficacy of carbamazepine compared to valproate semi-sodium, its induction of enzymes reducing antipsychotic levels, the mutually antagonistic effects of risperidone and paroxetine on mood, the side effects of Parkinsonism of both risperidone and paroxetine, and the side effects of hyperprolactinaemia, which can exacerbate osteoporosis, and weight gain of risperidone. At review two months later, no changes of any kind had been implemented. Following discussions amongst the treating team it was decided "the community nurse thinks there should be no changes to her medication as over the last 3–4 years she has been stable...she is 73 years old and not a young woman"

3 A 59 year old man with bipolar affective disorder and a recent TIA, taking 700 mg lithium daily (level 0.9), chlorpromazine 300 mg daily and 10 mg procyclidine daily. He complained of anxiety symptoms, restlessness and a tremor of several months' duration. He was advised to reduce and stop his chlorpromazine and procyclidine over a three month period, and reduce the dose of lithium to 600 mg daily. At review the patient had successfully stopped these medications and his tremor was much reduced. His GP had started a small dose of buspirone, and his anxiety and general mood were much improved. He was much more socially active and was attending further education.

4 A 55 year old lady, the wife of patient 3 above. Her GP referred her with addiction to sleeping tablets and mentioned that she stayed in bed all day. She was taking chlorpromazine 300 mg, stopped two weeks before being seen by ourselves, as she had begun to complain of worsening tremor, but when seen was still taking procyclidine 10 mg daily. At interview the patient gave a four year history of chronic anxiety and depression previous to which she had probably been dependent on alcohol for 11 years, consuming 70 units per week. Her depressive symptoms approached psychotic intensity and in addition she had orofacial dyskinesia. She was advised to stop procyclidine and to commence venlafaxine up to 225 mg daily: she had failed to respond to SSRIs previously. When seen four months later, the patient's husband said she was like a different woman: her depression had almost completely resolved, she was attending further education and had managed to give up smoking. Her GP had added a small dose of buspirone to her venlafaxine. She had successfully stopped her procyclidine and had no orofacial dyskinesia.

5 A 34 year old man with schizophrenia taking 10 mg risperidone daily i.e. greater than the recommended dose, and fluoxetine 20 mg daily: no indication for fluoxetine could be established. The patient had a BMI of 36 alongside poorly controlled positive symptoms, negative symptoms which had led to his losing his employment, and side effects of restlessness, gastrointestinal disturbance, blurred vision and abnormal involuntary movements alongside marked weight gain. The patient was advised to stop fluoxetine which was thought to be exacerbating his positive symptoms and abnormal movements, and responsible for gastro-intestinal disturbance. He was advised to substitute amisulpride at the low dose of 300 mg daily for the large dose of risperidone: this drug is associated with very little weight gain and is very effective for negative symptoms at such low doses, while maintaining efficacy for positive symptoms. At review the patient was taking 200 mg of amisulpride daily: his positive and negative symptoms were much improved, he was much more active and no longer complained of abnormal movements or gastro-intestinal disturbance. In addition, he reported much better memory and concentration.

6 A 73 year old lady taking venlafaxine 150 mg daily and 5 mg olanzapine at night. She had a history of recurrent depression but never any psychotic symptoms. Three years previously a consultant psychiatrist had advised reduction of the antipsychotic drug but this had not been implemented. The patient was not depressed at all but complained of having gained at least 7 lb weight on olanzapine: her BMI was 26. She was advised to stop this drug. At review four months later, the patient had stopped the olanzapine successfully: she had lost 7 lb in weight, and her BMI was 24. Furthermore the patient felt her energy levels were significantly improved, with less sedation and more capacity for physical activity.

## Discussion

Antipsychotic prescription is not rare in primary care patients: furthermore in this study over half was potentially problematic in terms of accepted prescribing standards, leaving aside the lack of diagnostic justification available in GP records. The situation in secondary care has been investigated using suboptimal prescribing criteria not dissimilar to our own [19]. It was found that nearly 46% of regimes were suboptimal: greater consultant contact was associated with better prescribing practice. These authors concluded that prescribing practices in real-world settings frequently deviated from evidence-based guidelines. We would add that this deviation may be substantially more extensive in primary and general secondary care compared to specialist secondary care, and would tentatively assume that the lack of consultant psychiatrist input may be a factor here. For instance another primary care audit of 170 patients prescribed atypical antipsychotics drugs found nearly all were subject to psychotropic polypharmacy, over a third had no licensed indication, 30% were over 75 years old, only half were monitored six monthly or more: half had not seen a consultant [6]. A population based observational study in primary care demonstrated a 16% increase in the use of antipsychotic drugs over a decade [14]. More than half of all first-time use was for depression, panic and anxiety disorder with less than 10% for psychosis: thioridazine, which was virtually withdrawn shortly after this study ended in 2000, was most commonly prescribed throughout.

Further research on atypical antipsychotic drug prescribing trends in primary care found a six-fold increase in five years in the West Midlands: rates of use varied three-fold within the region even when local population need was accounted for. In generalist secondary care medicine in Germany, it has been shown that a minority of prescriptions for antipsychotic drugs were for indications of psychosis, over half were for patients age 65 or older, and only 40% were given by psychiatrists: the rates of prescription had risen in parallel with a decrease in prescribing benzodiazepines [16].

A recent study of one sixth of the population of Italy reported that one in 50 elderly people were prescribed antipsychotic drugs during a single year, two-thirds being conventional drugs [21]. In nursing homes in the USA, 27.6% of residents were given antipsychotic treatment in 2000–2001: less than half received treatment following appropriate guidelines, and its effectiveness did not differ whether guidelines were followed or not [5]. A further German study [11] demonstrated that 6% of a population of 25 million were prescribed antipsychotic drugs at least once within a two and a half year period: again, most prescriptions were for conventional antipsychotic drugs, written by non-specialists. These authors expressed concern regarding the high frequency of psychotropic polypharmacy, and co-prescription of cardiovascular and metabolic treatments. Some of the atypical antipsychotic drugs may be particularly associated with cardiac and metabolic side effects.

A French utilization study has confirmed high rates of psychotropic polypharmacy alongside antipsychotic treatment, despite lack of evidence for the efficacy of such combinations [17]. By contrast, a study of private psychiatric practice in Switzerland demonstrated strong adherence to international guidelines, with low use of antipsychotic polypharmacy and psychotropic comedication [23].

There is no shortage of material advising against the practices which we, and others in the field, have encountered. Patients without schizophrenia and the elderly may be particularly liable to serious side effects of antipsychotic drugs [8]. Antipsychotic polypharmacy cannot be generally recommended, even in schizophrenia, because of lack of efficacy [13]: furthermore, it is associated with greater use of anticholinergic and benzodiazepine drugs [12]. Unlicensed prescribing of antipsychotics in dementia is not recommended: their use is associated with a threefold increased risk of serious cerebral cerebrovascular events [7]. It has been known for many years that non-psychotic subjects acutely exposed to conventional antipsychotic drugs may suffer persistent adverse effects, including dysphoria (subjectively unpleasant mood) for several weeks [15].

The patients in our study were not particularly symptomatic but were middle aged/elderly, and had a significant burden of motor side effects. Our patients' experience of worsening of symptoms and antipsychotic withdrawal syndromes is of particular concern. Re-emergence of symptoms for which the drug was originally prescribed has been described previously in a learning disabled population who discontinued thioridazine [18]. Deterioration was associated with longer period of treatment, and occurred regardless of whether the thioridazine was replaced with another antipsychotic drug.

More recent work has highlighted the gap between guidelines and utilization in the real world [26]. Economic and social conditions, specifically rapidly increasing economic growth, may be associated with rapidly increasing drug consumption [10]. If psychotropic medications are being prescribed for symptoms such as depression, insomnia and anxiety, which can be attributed as much or more to social and personal problems rather than genuine illness, doctors are in effect providing a medical solution where none is indicated. This excessive reliance on pharmacotherapy may bring with it irrational combinations of drugs in inadequate doses for long periods: clearly contrary to the principles of rational evidence based therapy.

Our limited results suggest stopping redundant antipsychotics reduces side effect burden. However, getting these patients seen, and implementing change, is very difficult indeed and not entirely without risk to patients' wellbeing. The obvious conclusion to be drawn is that the prescription of antipsychotic drugs, particularly in the long term, should be avoided in patients in whom these drugs are not indicated, or in whom benefits are likely to be marginal.

**Table 1:** Patients prescribed antipsychotic drugs in 7 practices in East Hull: failure of individual audit criteria

Total number of patients audited	52885	%
Patients prescribed antipsychotic drugs	534	1.01
Prescribed droperidol	0	0
Prescribed thioridazine	18	3.8
>1 antipsychotic	32	5.9
Antipsychotic + other psychotropic	172	32.2
No positive diagnosis	46	8.6
Anticholinergic drugs >3 months	64	12.0
Not reviewed within 12 months	16	3.0

**Table 2:** Patients failing single or multiple audit criteria: 'no positive diagnosis' excluded

Failing one criterion	39%
Failing two criteria	11%
Failing three criteria	4%
Failing 4 or more criteria	0.2%
Total failing one or more	52.4%
